# IDENTIFYING MULTIMORBIDITY RISKS IN OLDER ADULTS: TEN YEAR REVIEW USING THE RGA TOOL

**DOI:** 10.1093/geroni/igae098.4214

**Published:** 2024-12-31

**Authors:** Jinmyoung Cho, Wenjin Wang, Max Zubatsky, Allison Gibson, Marla Berg-Weger

**Affiliations:** Saint Louis University School of Medicine, Ballwin, Missouri, United States; Saint Louis University School of Medicine, Ballwin, Missouri, United States; Saint Louis University, St Louis, Missouri, United States; Saint Louis University, St Louis, Missouri, United States; Saint Louis University, St Louis, Missouri, United States

## Abstract

Approximately one in two older adults in the U.S. experiences multimorbidity, defined as the coexistence of two or more chronic diseases. The consequences of multimorbidity are significant, including increased vulnerability to acute illness, exacerbation of existing conditions, frequent hospitalizations, and elevated medical costs. Identifying the risk factors for multimorbidity in advance can guide healthcare service decision-making and help prevent adverse outcomes. This project utilized geriatric syndromes from the Rapid Geriatric Assessment (RGA) tool to assess the risk of multimorbidity. The RGA includes four geriatric syndromes: frailty, sarcopenia, geriatric anorexia, and cognitive decline. It was administered to a total of 16,615 individuals aged 65 years and over across Missouri from 2015 to 2024. Multimorbidity was defined as having five or more illnesses, which is one of frailty screening measures. Nearly 40% of the participants reported having five or more illnesses (37.3%). After controlling for age group, sex, race, and ethnicity, logistic regression analysis showed that the prevalence of sarcopenia (OR=3.807 [3.488 – 4.156], p <.001), geriatric anorexia (OR = 1.329 [1.224 – 1.443], p <.001), and dementia (OR=1.273 [1.158 – 1.401], p <.001) were significantly associated with having multimorbidity. This project provides evidence that the RGA is a valid screening tool for identifying multimorbidity across different practice settings and geographic regions of Missouri. The findings underscore the importance of early detection of geriatric syndromes to prevent further morbidity and disability among older adult populations.

